# Angiotensin-I-Converting Enzyme (ACE)-Inhibitory Peptides from Plants

**DOI:** 10.3390/nu9040316

**Published:** 2017-03-23

**Authors:** Ceren Daskaya-Dikmen, Aysun Yucetepe, Funda Karbancioglu-Guler, Hayrettin Daskaya, Beraat Ozcelik

**Affiliations:** 1 Department of Food Engineering, Faculty of Chemical and Metallurgical Engineering, Istanbul Technical University, Maslak, Istanbul 34469, Turkey; daskayac@itu.edu.tr (C.D.-D.); aysunyucetepe@gmail.com (A.Y.); karbanci@itu.edu.tr (F.K.-G.); 2 Department of Anesthesia and Reanimation, Bezmialem Vakif University Medical Faculty, Istanbul 34093, Turkey; hdaskaya@bezmialem.edu.tr; 3 BIOACTIVE Research & Innovation Food Manufacturing Industry Trade LTD Co., Maslak, Istanbul 34469, Turkey

**Keywords:** angiotensin-I-converting enzyme inhibitory activity, bioactive peptides, plant proteins, enzymatic hydrolysis, bioavailability, obesity, diabetes

## Abstract

Hypertension is an important factor in cardiovascular diseases. Angiotensin-I-converting enzyme (ACE) inhibitors like synthetic drugs are widely used to control hypertension. ACE-inhibitory peptides from food origins could be a good alternative to synthetic drugs. A number of plant-based peptides have been investigated for their potential ACE inhibitor activities by using in vitro and in vivo assays. These plant-based peptides can be obtained by solvent extraction, enzymatic hydrolysis with or without novel food processing methods, and fermentation. ACE-inhibitory activities of peptides can be affected by their structural characteristics such as chain length, composition and sequence. ACE-inhibitory peptides should have gastrointestinal stability and reach the cardiovascular system to show their bioactivity. This paper reviews the current literature on plant-derived ACE-inhibitory peptides including their sources, production and structure, as well as their activity by in vitro and in vivo studies and their bioavailability.

## 1. Introduction

Nutrition plays an important role in the prevention of cardiovascular diseases (CVD) such as atherosclerosis, coronary heart disease, stroke, and heart failure [1,2,3]. According to the World Health Organization (WHO), these diseases rank highest in the cause of global death [4]. The most important risk factor of CVDs is hypertension, a condition where the blood vessels have persistently raised pressure. Blood pressure drugs, especially inhibitors of the angiotensin-I-converting enzyme (ACE; EC 3.4.15.1) are generally used to regulate blood pressure in the renin-angiotensin system [3]. ACE is a catalyst in the production of the active hypertensive hormone angiotensin II from the inactive prohormone angiotensin I, and plays a role in degrading bradykinin, a vasodilator [5,6]. Active hypertensive bradykinin is formed from bradykininogen, and turns into inactive fragments through the actions of kinase II. In that way, constant blood pressure is conserved by the hypertensive peptide, angiotensin II, and the hypotensive peptide, bradykinin [6]. Due to the important roles of ACE in the regulation of blood pressure, the inhibition of this enzyme has been used to treat hypertension [7]. The first ACE inhibitors were isolated from the snake venom of *Bothrops jararaca* [8]. The drugs captopril, lisinopril, and enalapril were developed based on a snake venom peptide scaffold [9].

The effectiveness of the drugs could be different depending on medication. It has been reported that the effectiveness of the inhibitors on hypertensives were 40%–50% when used as a mono-therapy, and reached to 80%–90% when used with a diuretic [10]. However, some side effects of these inhibitors such as dry cough, taste disturbances and skin rashes from long term usage were reported [10,11]. Therefore, research has turned towards biological sources like plant extracts. It has been reported that the methanolic extracts of *Musa X paradisiaca* inhibited ACE by 68.63%–98.3% [12]. In another study, the ACE-inhibitory activity of citrus leaf extracts were reported in rats fed with palm oil heated five times [13]. In addition to plant extracts, food derived ACE-inhibitory peptides have been used as an alternative to synthetic drugs and are considered as the best known class of bioactive peptides [9]. Several studies have reported that food originating peptides could be used as an alternative ACE inhibitor with their low IC_50_ value to synthetic drugs [2,3,10,14,15,16]. The half maximal inhibitory concentration (IC_50_) value is the amount of inhibitor required to inactivate 50% of ACE activity under the experimental conditions [17]. Among the food originating sources, plant proteins and microalgae species show potential as they can be produced in a cost-efficient and environmental sustainable manner when compared to animal sourced proteins [18]. Indeed, peptides from plant sources may be preferred by vegetarians. Different types of plants have been used to obtain ACE-inhibitory peptides such as wheat, peas, mushrooms, soybeans, walnuts, date seed flour, bitter melon seeds and spinach [6,11,14,17,19,20,21,22,23,24,25,26,27]. Among them, soybean based peptides have been most commonly used [1,23,24,28,29]. However, recent research has been conducted to investigate novel peptides from different sources [30,31,32,33] and waste [34,35,36] to produce added-value products. A summary of the studies on ACE-inhibitory peptides derived from plants is provided in Table 1.

ACE inhibition by the peptides can be competitive or non-competitive enzyme inhibition [15,28,37]. Competitive enzyme inhibition is defined as the interaction of the inhibitor with the active enzyme sites to prevent substrate binding [38]. Noncompetitive enzyme inhibition refers to the situation where the inhibitor molecule has binding affinity for both the free enzyme and the enzyme–substrate complex [32]. Jang et al. [6] reported that all of the purified ACE inhibitors from the mushroom *Pleurotus cornucopiae* were non-competitive inhibitors. Additionally, Shi et al. [38] reported that the peptides from peanut bound competitively with the substrate at the active site of ACE and showed a competitive inhibitor pattern. To determine the inhibition pattern of peptides (competitive or non-competitive) on ACE, Lineweaver–Burk plots [6,38,39] and/or molecular docking [32] can be used. The type of bonding between the ACE inhibitor and enzyme can be elucidated by molecular docking. Sornwatana et al. [32] showed three types of binding interactions including hydrogen bonding, hydrophobic, and ionic interactions in the chebulin (a peptide obtained from fruit proteins) and ACE.

The aim of this manuscript is to review the recent studies on plant-derived ACE-inhibitory peptides including their sources, production, and structure, and to discuss their activity by in vitro and in vivo studies and their bioavailability.

## 2. Angiotensin Converting Enzyme and Its Inhibition Mechanism

ACE is a glycoprotein which contains carbohydrate moiety composed of mannose, galactose, fructose, N-acetylneuraminic acid, and *N*-acetyl-glucosamine [5]. Depending on the carbohydrate moiety, the molecular mass of ACE can be different between 130 and 170 kDa. ACE consist of two domains, which are *N*-domain and C-domain and they each contain a zinc cofactor binding active site [45]. Therefore, ACE can be inhibited by metal chelating agents [46]. The most effective inhibitors were reported as EDTA; CdBr2; angiotensin II; bradykinin; and a pentapeptide, l-pyroglutamyl-l-lysyl-l-tryptophyl-l-alanyl-l-proline, a component of *Bothrops jararaca* venom [47]. ACE is a member of the M2 family metallopeptidase [48] and have an important role in hypertension.

The regulation of blood pressure is controlled by the renin-angiotensin system or the renin-angiotensin-aldosterone system. The body secretes a proteolytic enzyme called renin from the kidneys as a defensive mechanism to decrease blood pressure. The secreted renin catalyzes the conversion of angiotensinogen, which is continuously secreted from the liver, to an inactive decapeptide called angiotensin I (Asp-Arg-Val-Tyr-Ile-His-Pro-Phe-His-Leu). The inactive angiotensin I is then converted to active octapeptide angiotensin II (Asp-Arg-Val-Tyr-Ile-His-Pro-Phe) by an ACE enzyme found in plasma. Angiotensin is forty times more potent compared to a noradrenaline hormone and accepted as one of the strongest vasoconstrictors [49]. In addition to the blocking effect in angiotensin II synthesis, ACE inhibitors also cause vasodilatation and a decrease in blood pressure via the inhibition of the kininase 2 enzyme and decreasing bradykinine destruction [50].

## 3. Production of ACE-Inhibitory Peptides from Plants

**Figure 1:**
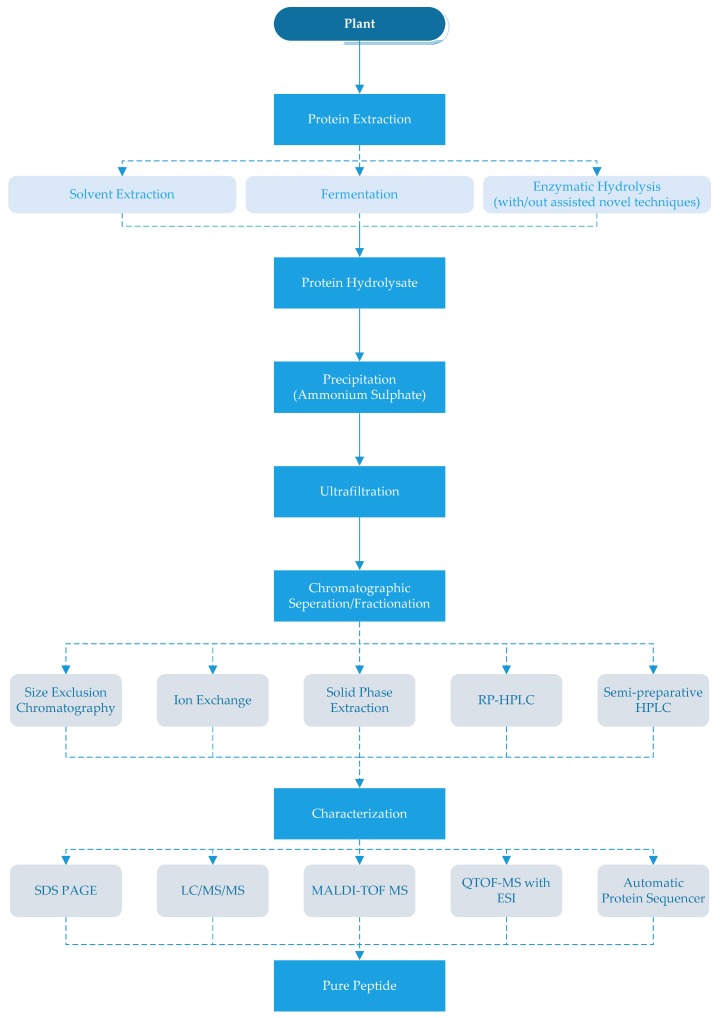
Production of angiotensin-I-converting enzyme (ACE) inhibitory peptides. RP- HPLC: reverse-phase high performance liquid chromatography SDSPAGE: sodium dodecyl sulfate-polyacrylamide gel electrophoresis, LC/MS/MS: liquid chromatography-tandem mass spectrometry, MALDI-TOF MS: matrix-assisted laser desorption ionisation-time of flight mass spectrometry, QTOF-MS with ESI: quardrupole time-of-flight mass spectrometer (Q-TOF-MS) with an electro-spray ionization (ESI).

There are different methods to obtain ACE-inhibitory peptides from plants that are summarized in Figure 1. The peptides can be extracted from plant proteins by using solvents and/or proteolytic enzymes [6,17,51]. Methanol, ethanol and water have been used as solvents and their extraction yield has varied according to the plants and type of solvent in a mixture [6,25]. Jang et al. [6] extracted the mushroom peptides with a mixture of methanol and water in varying ratios and reported that the water extracts had higher ACE-inhibitory activity (78.0%) compared to the methanol extracts (55.0%).

Even though solvents can be used solely to obtain ACE-inhibitory peptides, enzymes can also be included. Thewissen et al. [17] extracted peptides with 70% ethanol and extracts were hydrolyzed with trypsin, ficin, or thermolysin.

Several different proteolytic enzymes are reported to hydrolyze proteins such as alcalase, flavourzyme, thermolysin, trypsin, chymotrypsin, pepsin, papain, neutrase, and bacterial and fungal proteases [11,17,51,52]. Regardless of the enzyme used, temperature, hydrolysis time, and enzyme to protein ratio are considered crucial factors in the production of peptide with ACE-inhibitory activity [10,26,35,51]. Due to the specificity of the proteolytic enzyme to substrate, the peptide composition of hydrolysates, and therefore ACE-inhibitory activities change [52,53]. Since ACE-inhibitory peptides are generally short chained, enzymes should have a high hydrolysis degree [54,55]. Optimum temperature and pH can be different depending on the enzyme, for example, 50 °C and pH 9.0 for alcalase [56], 50 °C and pH 6.0 for flavourzyme [57], 50 °C and pH 8.0 for thermolysin [35], and 65 °C and pH 7.0 for papain [58]. Alcalase and thermolysin are capable of producing relatively higher ACE-inhibitory active peptides than other proteolytic enzymes [14,26,35,36,40,51,57]. The peptides with rich hydrophobic amino acid content such as Ala, Val, Leu, Ile, Phe, Pro, Trp, and Met at C-terminal residue can be obtained by alcalase due to its cleavage preference [59]. As far as thermolysin is concerned, peptides with hydrophobic N-terminal residue such as Trp, Tyr, Phe, Ile, Leu, Val, Ala or Met are known to be important for the potency of peptides [60,61].

Additionally, pepsin, trypsin and α-chymotrypsin are other important enzymes that have been utilized in the gastrointestinal simulation of peptide digestion [2,28,62]. Pepsin has broad cleavage specificity with a preference for peptides containing linkages with aromatic or carboxylic l-amino acids. It preferentially cleaves C-terminal to Phe and Leu, which are important amino acids for the ACE-inhibitory capacity of peptides [5,32]. α-chymotrypsin has cleavage of peptide bonds with bulky side chains and non-polar amino acids [63]. The peptides with C-terminus amino acids such as Val, Ala, Leu, Pro, Tyr, Phe, His and Trp can be obtained by α-chymotryptic hydrolysis [64]. Trypsin, on the other hand, cleaves the peptide after Lys or Arg [65].

In some studies, the combination of enzymes are used to increase the level of potential ACE-inhibitory peptides due to enzyme specificity [14,66]. Indeed, pepsin, α-chymotrypsin and trypsin were combined to simulate the gastrointestinal digestion of food proteins in humans [9,11,15]. It has been reported that soybean protein digested only by thermolysin and thermolysin–pepsin combination had similar ACE-inhibitory activities, with an IC_50_ value of 51.8 µg/mL and 53.6 µg/mL, respectively. However, a higher IC_50_ value of 115.6 µg/mL has been obtained by using a triple combination of thermolysin, pepsin and trypsin, indicating that further digestion by trypsin can influence the ACE-inhibitory activity of soybean [11]. Furthermore, a combination of two or more enzymes may show higher ACE-inhibitory activity than single enzymatic treatment. In a study discussed in Reference [14], a high ACE-inhibitory activity was obtained with an alcalase and thermolysin mixture showing an IC_50_ value of 530 µg/mL than individual enzymes.

Several proteolytic enzymes obtained from different sources and commercial enzymes which were used to obtain ACE-inhibitory peptides are mesophilic with optimum temperatures ranging from 30–65 °C [54]. However, cold active enzymes obtained from cold adaptive microorganisms are active at lower temperature, therefore, energy saving and prevention of undesirable by-product formation could be possible [54,67]. Additionally, it has been reported that cold active proteolytic enzymes have higher specificity than mesophilic ones [54,67,68,69,70]. As an alternative to soluble enzymes, immobilized enzymes can be used in ACE-inhibitory peptide production and have several advantages in terms of process conditions [71] as they do not need to be inactivated by heat or acidification [72]. Different from soluble enzymes, immobilized enzymes can be easily recovered and reused [73].

Research has been conducted using novel techniques such as high pressure (HP), ultrasound, and microwave to obtain high yields in the enzymatic production of ACE-inhibitory peptides [16,19,74]. Garcia-Mora et al. [41] showed that HP assisted proteolysis with different enzymes increasing the ACE-inhibitory activity of lentil hydrolyzates by obtaining more peptides with short chains (<3 kDa). It has been concluded that HP promoted the release of bioactive sequences most likely due to the higher accessibility of enzymes to the substrate and the exposure of new target residues [41]. Similar to HP treatment, ultrasound techniques have also been used as an assistant to the enzymatic process [19,75]. More peptides with a C-terminal hydrophobic amino acid residue, which have an important role on ACE inhibition, can be obtained by ultrasonic treatment, as this pretreatment causes more hydrophobic groups and regions inside the molecules to be exposed outside [75]. In another study, Otag and Hayta [74] investigated the effect of microwaves on ACE-inhibitory activity of peptides derived from chickpea with enzymatic hydrolysis and found higher ACE-inhibitory activity in microwave treated samples (increased by 4.5%).

In addition to the methods explained above, ACE-inhibitory peptides can be obtained through fermentation using cultures with proteolytic activities [76]. Lactic acid bacteria (LAB) are generally used as a starter culture for ACE-inhibitory peptide fermentation [21,76,77,78,79]. However, studies show that microorganisms other than LAB such as *Bacillus* spp. [22,34] *Staphylococcus vitulus* [80], *Saccharomyces cerevisiae* [5,21,81], *Debaryomyces hansenii* [82], *Mucor* spp. [83], and *Aspergillus* spp. [84] can be used as starter cultures.

Starter cultures, fermentation conditions and inoculum levels play important roles in ACE-inhibitory peptide production [21,79,80]. Shu et al. [79] reported that ACE-inhibitory activity increased with fermentation time in milk fermented by *Lactobacillus plantarum*, as reported in mao–tofu fermented by *Mucor* spp. [83] and peas fermented with *L. plantarum* [21]. As inoculum level is important, it has been reported that ACE-inhibitory activity decreased with inoculum level, probably due to the decomposition of ACE-inhibitory peptides by a high microbial load [79]. In addition to the fermentation conditions and inoculum level, the strain and the type of co-culture used also play an important role in ACE-inhibitory peptide production. It has been reported that fermentation with co-cultures of *Pichia kudriavzevii*, *Enterococcus faecalis*, and *L. plantarum* produced ACE-inhibitory peptides showing low IC_50_ values (30.63 µg/mL) with low bitterness [85].

## 4. Purification of Peptides and Sequencing

After the production of ACE-inhibitory peptides, different separation and purification techniques should be applied or may be required to obtain pure products. The first step of purification is generally for the hydrolyzate to be precipitated with ammonium sulfate and then desalted. Since ACE-inhibitory peptides are generally small, the hydrolyzates are generally filtered through a 3000 Da or 5000 Da cut-off membrane [3,6,32]. In the next step, different chromatographic techniques such as size exclusion chromatography, immobilized metal-affinity chromatography (IMAC), ion exchange chromatography and/or reversed-phase HPLC (RP-HPLC) have been used to remove compounds other than peptides [6,17,39].

Size exclusion chromatography with resins that can separate smaller molecules can be used for the chromatographic separation/fractionation of the peptide hydrolysates [34,39]. As an alternative to size exclusion chromatography, anion-exchange or cation-exchange chromatography can be used by selecting suitable mobile phases [15,25]. Indeed, immobilized metal-affinity chromatography (IMAC) can be used to purify peptides with an affinity to metal ions from hydrolyzates. The IMAC fraction may be rich in histidine and hydrophobic AA (Pro, Val, Ile, Leu and Phe) [17]. Further purification of peptides may be carried out by semi-preparative HPLC [9,38,54,86,87]. Since ACE-inhibitory peptides generally contain hydrophobic amino acids, higher retention occurs in the hydrophobic chromatography column [88]. Each of these techniques could be used solely or in combination (as shown in Table 1).

The purification of peptides and/or enzymatic hydrolysis could increase the ACE-inhibitory activity of peptides [33,46]. It has been reported that a reduction was observed on the IC_50_ value of peptides from mushrooms, which were 310 µg/mL for water extract and 40 µg/mL at the end of the last purification step, RP-HPLC [39]. Similarly, Jang et al. [6] showed that the IC_50_ value of peptides from mushrooms decreased from 6000 µg/mL (water extraction) to 460 µg/mL at the end of purification. The reduction of the IC_50_ value of peptides depending on purification could be related to the protein concentration of the peptides. Liu et al. [25] showed that the ACE inhibition rate of peptides increased from 38.5% to 82.8% with the protein concentration of the peptides (15–90 µg/mL) due to purification.

After purification, the collected fractions should be freeze-dried and examined for their ACE-inhibitory activity [25,26]. Next, the ACE-inhibitory peptides can be identified and characterized by sodium dodecyl sulfate-polyacrylamide gel electrophoresis (SDS-PAGE), mass spectrometry and protein sequencing to determine molecular mass, amino acid composition and sequence as shown in Table 1 [14,26,31,32,34,38,41,43].

## 5. Structural Characteristics/Structure Activity Relationship

**Figure 2:**
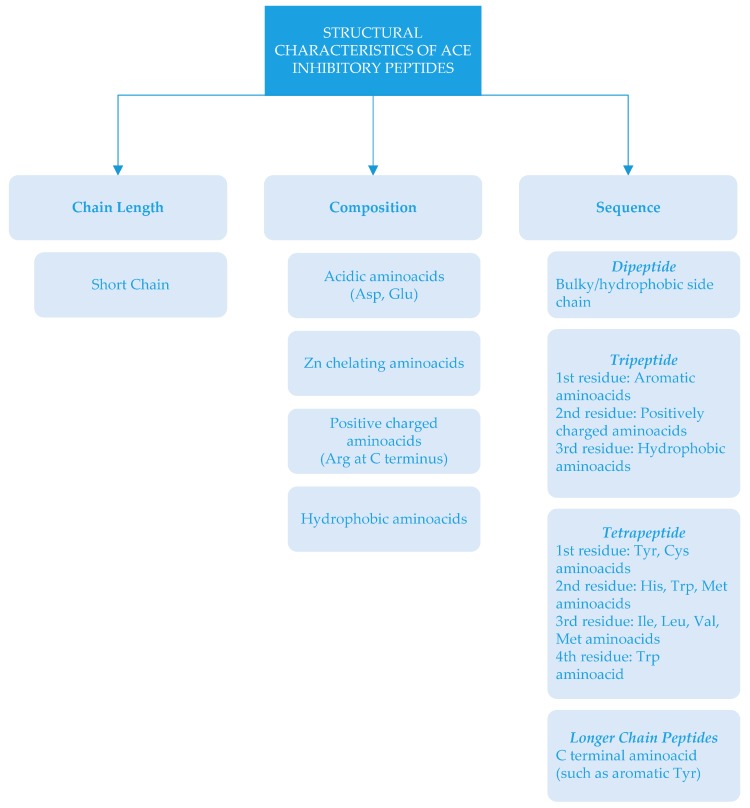
Structural characteristics of angiotensin-I-converting enzyme (ACE) inhibitory peptides.

There is a relationship between the structure of peptides and ACE inhibition. Depending on chain length, amino acid composition and sequences, ACE-inhibitory activity can be different [16,46]. Structural characteristics of ACE-inhibitory peptides (which have important roles in ACE-inhibitory activity) are summarized in Figure 2.

ACE-inhibitory peptides are generally short chain peptides with 2–12 amino acids and crystallography studies show that large peptides cannot bind to active sites of ACE [89]. In some cases, long chain peptides could have ACE-inhibitory activity [19]. In some cases, the amino acid type could be more important than the length of the peptide. This could be related to amino acid composition, since peptides contain highly acidic amino acids (Asp and Glu) that may cause a net negative charge. The interaction of negatively charged peptides with ACE could chelate zinc atoms, which are necessary for enzyme activity [46]. ACE-inhibitory peptides generally consist of specific amino acid residues at the C- end and/or N- end [2]. It has been reported that the presence of tyrosine, phenylalanine, tryptophan, proline, lysine, isoleucine, valine, leucine, and arginine in peptides have a strong influence on ACE binding [5,11,15,90]. Due to the reduction of ACE inhibition—depending on the removal of Arg residue at the C-terminus [5,91]—it has been stated that amino acids with positive charges at the C-terminus also influence the inhibitory effects of the peptides [15]. There are different sequences of ACE inhibitors ranging from dipeptides to oligopeptides [6]. These dipeptides contain amino acids with bulky and hydrophobic side chains. In the case of tripeptide, the structural feature was reported as an aromatic amino acid at the first residue, a positively charged amino acid at the second residue, and a hydrophobic amino acid at the third residue [92]. The structure of ACE-inhibitory tetrapeptides has been expressed as Tyr and Cys present at the first position; His, Trp, and Met at the second position; Ile, Leu, Val and Met at the third position; and Trp at the fourth position [93]. As far as peptides with longer chains are concerned, the inhibition effect has been related to C-terminal amino acids [2].

ACE-inhibitory peptides are generally short chain peptides with 2–12 amino acids and crystallography studies show that large peptides cannot bind to active sites of ACE [89]. In some cases, long chain peptides could have ACE-inhibitory activity [19]. In some cases, the amino acid type could be more important than the length of the peptide. This could be related to amino acid composition, since peptides contain highly acidic amino acids (Asp and Glu) that may cause a net negative charge. The interaction of negatively charged peptides with ACE could chelate zinc atoms, which are necessary for enzyme activity [46]. ACE-inhibitory peptides generally consist of specific amino acid residues at the C- end and/or N- end [2]. It has been reported that the presence of tyrosine, phenylalanine, tryptophan, proline, lysine, isoleucine, valine, leucine, and arginine in peptides have a strong influence on ACE binding [5,11,15,90]. Due to the reduction of ACE inhibition—depending on the removal of Arg residue at the C-terminus [5,91]—it has been stated that amino acids with positive charges at the C-terminus also influence the inhibitory effects of the peptides [15]. There are different sequences of ACE inhibitors ranging from dipeptides to oligopeptides [6]. These dipeptides contain amino acids with bulky and hydrophobic side chains. In the case of tripeptide, the structural feature was reported as an aromatic amino acid at the first residue, a positively charged amino acid at the second residue, and a hydrophobic amino acid at the third residue [92]. The structure of ACE-inhibitory tetrapeptides has been expressed as Tyr and Cys present at the first position; His, Trp, and Met at the second position; Ile, Leu, Val and Met at the third position; and Trp at the fourth position [93]. As far as peptides with longer chains are concerned, the inhibition effect has been related to C-terminal amino acids [2].

In addition to inhibition capacity, structural characteristics play an important role in the bitterness of peptides. The bitterness of ACE-inhibitory peptides have been considered an important problem for their incorporation into food items [16]. It has been reported that the location of bulky groups and hydrophobic amino acids play important roles in the bitter taste of ACE-inhibitory peptides [94,95].

A novel peptide from walnut protein hydrolyzate was isolated, purified and its sequence determined as Trp-Pro-Glu-Arg-Pro-Pro-Gln-Ile-Pro. The ACE-inhibitory activity of the peptide was related to the hydrophobic amino acid content, Trp and Pro. Indeed, the positively charged amino acids and aromatic amino acids could contribute to the ACE-inhibitory activity of peptides [25]. In another study [26], several ACE-inhibitory peptides were purified from the proteolytic hydrolyzate of bitter melon seed proteins and the sequence of the strongest inhibitory peptide was reported as Val-Ser-Gly-Ala-Gly-Arg-Tyr. The aliphatic and hydrophobic residues at its N-terminus, a basic arginine residue in the middle, and an aromatic tyrosine at the C-terminus could make that peptide superior in terms of ACE-inhibitory activity [34]. In a recent study, a short peptide with the sequence Tyr-Ser-Lys was obtained from rice bran protein and the molecular docking study on this peptide showed that the peptide formed very strong hydrogen bonds with the active sites of ACE [31].

Although the relationship between ACE-inhibitory activity and peptide structure has not been fully elucidated, it is possible to conclude that the inhibitory potential of peptide depends on its structural and compositional characteristics.

## 6. Activity of ACE Inhibitors Derived from Plants

### 6.1. In Vitro Studies

There are different methods to measure ACE-inhibitory activity using various substrates, media, and analytical techniques [17,32,41,47]. Among them, the most used method was developed by Cushman and Cheung in 1971 [47], based on the determination of the concentration of hippuric acid (HA) by spectrophotometry at 228 nm after ethyl acetate extraction, which is formed from hippuryl-histidyl-leucine (HHL) by the action of ACE [47]. Even though this assay is simple and economical, there have been some reported limitations. Interference from unhydrolyzed HHL, which also absorbs strongly at 228 nm, may cause overestimation of ACE activity [96]. Indeed, when applied to complex peptide mixtures, interfering molecules may also cause incorrect measurements [3]. In terms of substrate, *N*-(3-(2-furyl)acryloyl)-l-phenylalanylglycylglycine (FAPGG) or 3-hydroxybutyrylglycyl-glycyl-glycine (3HB-GGG) can be used as an alternative to HHL [17,97]. Furthermore, different analytical techniques can be used to measure ACE activity such as a fluorescence-based protocol [41,98] or chromatographic method [3,11,99]. Fluorescence-based protocols are based on the measurement of the generated fluorescence every minute for 30 min at emission and excitation wavelengths of 355 and 405 nm, respectively, in a microplate fluorometer [41,98].

A chromatographic method was based on the separation of HHL and HA by using HPLC with mixtures of trifluoroacetic acid (TFA)-acetonitrile and TFA-water as the mobile phase [11,99] and was quantified with a diode array detector (DAD) [3,42].

In addition to analytical methods, it is possible to predict the ACE-inhibitory activity of a peptide by using the quantitative structure–activity relationship (QSAR) model [2,11,100]. The sequences of the peptides obtained from soy protein have been identified with LC-MS/MS and the IC_50_ value of the ACE-inhibitory activity of peptides was predicted by the QSAR model based on its composition and sequence, and it has been reported that the predicted IC_50_ values were close to the experimental IC_50_ values [11].

### 6.2. In Vivo Studies

The ACE-inhibitory activities of the peptides could be evaluated by in vivo assays as well. However, the lack of correlation between the in vitro ACE-inhibitory activity and the in vivo action should be noted. While in vivo studies offer the advantage of an intact system that allows physiological transformations and metabolic interactions that could change the efficacy of the peptides, in vitro studies provide results on the capability of the ACE-inhibitory activities of peptides [16].

The ACE-inhibitory activities of peptides with in vivo studies are generally investigated by a periodic blood pressure measurement in spontaneously hypertensive rats (SHRs) after intravenous or intraperitoneal injection and oral gavage. In a study on peptides derived from hemp-seed, the blood pressure lowering effects of peptides were investigated by systolic blood pressure measurement following oral administration to SHR in comparison with a synthetic drug used as a positive control and a negative control (saline) [33]. Antihypertensive effects of the peptide from an edible mushroom at a dosage of 1 mg/kg per body weight were determined following oral administration to SHRs [39]. In a recent study, a novel peptide from bitter melon seed proteins at 2 mg/kg body weight showed effects of lowering blood pressure [26]. In another study, the maximal reduction in arterial blood pressure of pea permeate was determined as 32% after femoral vein injection. Higher reduction ratio by a synthetic drug (captopril) was also reported in the same study [101]. Similarly, captopril showed stronger antihypertensive activity than peanut peptide after oral feeding [38]. Furthermore, peptides from plant proteins also have potential as antihypertensive agents. Although compatible results have generally been obtained both in in vitro and in vivo studies, some differences have been reported between the IC_50_ and blood pressure lowering effect in SHRs [15,33]. Functional experiments (translational/integrated research) are required to identify what the effects of peptides at the cellular level means for the entire system [102].

## 7. Bioavailability of ACE Inhibitor Peptides

Bioavailability is a crucial factor in obtaining health benefits from a food component [103]. The bioavailability of a component is controlled by their liberation from food items, chemical transformation during the gastrointestinal tract, solubilization in the intestinal fluids, permeation through the intestinal cell monolayer, and efflux from epithelium cells [104]. ACE-inhibitory peptides must remain active during gastrointestinal digestion and absorption and reach the cardiovascular system to show their bioactivity [105]. Due to gastrointestinal digestion, the bioavailability of the obtained peptide and therefore ACE-inhibitory activity may be affected by amino acid composition and the chain length of the peptides. Proline- and hydroxyproline-containing peptides and tripeptides containing the C-terminal proline-proline have been reported as resistant to degradation by digestive enzymes [106]. Indeed, the chain length of the peptide could be critical to resisting gastrointestinal digestion. Oligopeptides are more easily hydrolyzed by various protease and brush border membrane peptidases, and are more difficult to be absorbed intact through the intestine [107].

Pepsin, trypsin, α-chymotrypsin and pancreatin are often used to investigate whether ACE-inhibitory peptides can resist gastrointestinal digestion [11,62]. The stability and absorption of the peptides obtained from bitter melon seed proteins during gastrointestinal digestion were simulated using pepsin and trypsin enzymes by Priyanto et al. [26]. It has been reported that the stability of the peptides differed depending on the simulated gastrointestinal digestion. Results of the study showed a reduction on the ACE-inhibitory activity of the peptide mixture (from 50% to 28%), while another peptide mixture was reported as unaffected by in vitro gastrointestinal digestion [26]. Similarly, Jimsheena and Gowda [9] obtained ACE-inhibitory peptides from the peanut protein arachin, by simulating gastric digestion using pepsin, trypsin, chymotrypsin and pancreatin enzymes. Among the obtained peptides, the peptide with the sequence Asn-Ala-Gln-Arg-Pro was reported as the most effective due to its short chain length and Pro at C-terminal structural characteristics [9]. In another study, Boschin et al. [3] reported that ACE-inhibitory activity could be different depending on gastrointestinal digestion due to the cleavage sites of enzymes. They treated lupin proteins with pepsin that enabled them to obtain peptides with hydrophobic and aromatic amino acids as well as peptides with lower IC_50_ values (mean: 186 µg/mL). The proteins were treated with trypsin, which cleaved the peptidic bonds involving basic amino acids, and pepsin, after which an increase on the IC_50_ values of the peptides (198 µg/mL) were observed. Indeed, they treated the lupin proteins with Corolase PP, a patented complex which includes a porcine pancreatic proteolytic preparation to simulate gastrointestinal digestion and have obtained peptides with moderate IC_50_ values (mean: 533 µg/mL) [3].

In addition to enzymatic methods, Caco-2 cells derived from a human colon carcinoma (similar to intestinal epithelium cells) can be used to predict the intestinal permeability of bioactive molecules [37,108,109,110,111]. Megias et al. [112] investigated the stability of sunflower peptides by using Caco-2 cell extracts and reported a 60% reduction on the ACE-inhibitory activity of hydrolyzates at the end of 2 h incubation with Caco-2 cell extracts. However, the ACE-inhibitory peptide (Phe-Val-Asn-Pro-Gln-Ala-Gly-Ser) obtained by hydrolysis with pepsin and pancreatin was found just as stable against Caco-2 cells extracts [112]. Although the stability of the ACE-inhibitory peptide using Caco-2 cells has been investigated, these studies are limited. Most studies on the bioavailability of ACE-inhibitory peptides using Caco-2 cells have been focused on milk [108,113] or egg [107,114] based peptides.

Enhancement of the stability, delivery and bioavailability of bioactive peptides could be achieved by encapsulation applications. Encapsulation offers the conversion of sensitive materials to stable components against gastrointestinal digestion and food processing conditions [16,115,116,117]. Ruiz et al. [118] investigated the stability of ACE-inhibitory activity of bean peptides, which were encapsulated with a blend of Delonix regia carboxymethylated gum/sodium alginate. They found 16% and 60% release of protein at gastric and intestinal simulation conditions, respectively. Furthermore, the protein maintained the IC_50_ of 2900 µg/mL [118]. In another study, Yea et al. [115] produced nanoliposomes containing ACE-inhibitory peptides derived from winged bean seeds. It was reported that the incorporation of hydrophilic proteolyzate into hydrophobic liposomes was successfully achieved. Indeed, nano-sized particles showed good storage stability over 8 w at 4 °C [115]. According to our literature survey, there are still limited studies on the encapsulation of plant-based ACE-inhibitory peptides and future study in this area is needed to develop encapsulated peptides derived from plants.

## 8. Conclusions and Future Trends

Nutrition plays an important role in human health and in the prevention of many chronic diseases. It has been known that there is a relationship between hypertension, obesity, and diabetes. Hypertension in diabetes and obesity can increase the risk of morbidity and mortality. From this point of view, nutrition is even more important. Studies on bioactive peptides with antihypertensive activity further show the importance of nutrition.

Although there have been several studies on the effects of ACE-inhibitory peptides, most of the studies have been conducted on milk peptides. However, the consumer trend towards vegetarianism in recent years has meant that plant sources have gained more importance. In this context, more studies should be done on novel food sources like endemic, medicinal and aromatic plants and the production, purification and characterization of ACE-inhibitory peptides derived from these sources. In addition to these novel sources, plant waste and sub-products could be utilized to produce value-added commodities. Future studies should be conducted in these areas as well as on in vivo assays for ACE-inhibitory activity.

Additionally, scale-up studies are also necessary to produce peptides for the food and pharmaceutical industries. The studies on ACE-inhibitory peptides are generally performed at a lab scale. However, in order to industrialize these peptides, scale-up/pilot-plant scale studies are required. There are some studies on the pilot-plant scale production of ACE-inhibitory peptides from different types of plant sources (like peanut meal, corn wet milling byproducts and alfalfa white protein) [119,120,121]. As the industrialization of the ACE-inhibitory peptides is important as an alternative to synthetic drugs, more scale up studies need to be performed. Depending on these studies, it will be possible to use ACE-inhibitory peptides from plant sources as nutritional supplements and/or to incorporate them into other food products. As far as incorporation of peptides into food products is concerned, the bitterness of ACE-inhibitory peptides should be considered [16,122]. Different techniques have been developed to remove the bitter taste from ACE-inhibitory peptides, and encapsulation of the peptides is the most favorable technique used to mask the bitter taste of the peptides [16]. However, ACE-inhibitory peptides with low bitterness could be produced through co-culture fermentation [85]. Another strategy to eliminate the bitter taste of ACE-inhibitory peptides is the debittering of peptides by exo-peptidase [123]. Most studies on ACE-inhibitory peptides from plant sources have been focused on their production and characterization; however, it needs to be noted that the bitter taste of these peptides is also important to eliminate as far as their utilization in foods are concerned. In conclusion, the stability and bioavailability of the peptides obtained from novel plant sources and their incorporation into functional foods and nutraceuticals should be investigated in future studies.

## Figures and Tables

**Table 1:** Production, purification methods and characterization of angiotensin-I-converting enzyme (ACE)-inhibitory peptides derived from plants.

Substrate	Production Method	Purification Method	IC_50_ Value	Sequencing and Molecular Mass Determination	Peptide Sequence and Molecular Weight	Reference
Mushroom (*Tricholoma giganteum*)	Solvent extraction and enzymatic hydrolysis	Ultrafiltration (UF), size exclusion chromatography (SEC) with Sephadex G-25 column chromatography, and reverse-phase high performance liquid chromatography (RP-HPLC)	Water extract: 310 µg/mL	Protein sequencer	Gly-Gln-Pro 301 Da	[39]
			UF: 280 µg/mL			
			SEC: 240 µg/mL			
			RP-HPLC: 40 µg/mL			
Mushroom (*Pleurotus cornucopiae*)	Water and methanol extraction	UF, SEC with Sephadex G-25 column, solid phase extraction (SPE), strong cation exchange (SCX) solid phase extraction, RP-HPLC	Water extract: 6000 µg/mL	Liquid chromatography tandem mass spectrometry (LC-MS/MS)	Fr 1: Arg-Leu-Pro-Ser-Glu-Phe-Asp-Leu-Ser-Ala-Phe-Leu-Arg-Ala (1622.85 Da); Fr 2: Arg-Leu-Ser-Gly-Gln-Thr-Ile-Glu-Val-Thr-Ser-Glu-Tyr-Leu- Phe-Arg-His (2037.26 Da)	[6]
			UF: 5300 µg/mL			
			SEC: 3860 µg/mL			
			SCX: 1500 µg/mL			
			RP-HPLC:			
			Fr 1: 460 µg/mL			
			Fr 2: 1140 µg/mL			
Potato	Enzymatic hydrolysis with alcalase, neutrase and esperase	UF (3, 5 and 10 kDa cut off), SPE, HPLC	18–86 µg/mL	Matrix-assisted laser desorption ionization (MALDI)-time of flight (TOF) mass spectrometry (MS)	704–850 Da	[40]
Wheat	Solvent extraction and enzymatic hydrolysis	Immobilized metal-affinity chromatography and semi-preparative RP-HPLC	20 µg/mL	-	-	[17]
Soybean	*Lactobacillus casei* spp. *pseudoplantarum* fermentation	Semi-preparative HPLC	17.2 µg/mL^2^	Protein sequencer	N-terminal of the peptide: Leu-Ile-Val-Thr-Gln	[29]
	Enzymatic hydrolysis with thermolysin, pepsin and trypsin	RP-UPLC	Predicted by QSAR modelling based on peptide sequences: 3.4–470.7 µM	Reverse-phase ultra performance liquid chromatography tandem mass spectrometry (RP-UPLC-MS/MS)	12 dipeptide, 10 tripeptide, 7 tetrapeptide, 4 pentapeptie, 1 hexapeptide (200–600 Da)	[11]
Terminalia chebula Tree	Enzymatic hydrolysis with pepsin	Filtration (3–kDa cut off), RP-HPLC, sodium dodecyl sulfate-polyacrylamide gel electrophoresis (SDS- PAGE) and nano-LC-MS/MS	100 µM	Nano-liquid chromatography tandem mass spectrometry (Nano-LC-MS/MS)	Asp-Glu-Asn-Ser-Lys-Phe 738.5 Da	[32]
Lentil	HP assisted proteolysis with different proteolytic enzymes	UF (3–kDa cut off), SPE	-	MALDI TOF/TOF MS/MS	13 different peptides (1105–2614 Da)	[41]
Walnut	Enzymatic hydrolysis with proteinase	UF (3–kDa cut off), SEC with Sephadex G-15 and anion exchange chromatography, and HPLC	25.67 μg/mL	MALDI TOF MS	Trp-Pro-Glu-Arg-Pro-Pro-Gln-Ile-Pro 1033.42 Da	[25]
Tomato waste	*Bacillus subtilis* fermentation		8200 µg/mL^2^	MALDI TOF MS	500–800 Da	[34]
Rice bran	Enzymatic hydrolysis with trypsin	UF (different cut off; <4 kDa, 4–6 kDa, >6 kDa), SEC with Sephadex G-25, RP-HPLC	76 µM	Quardrupole time-of-flight mass spectrometer (Q-TOF-MS) with an electro-spray ionization (ESI) (Q-TOF-MS with ESI)	Tyr-Ser-Lys 395 Da	[31]
Apricot kernel	Enzymatic hydrolysis with different proteolytic enzymes	UF (1 and 5 kDa MWCO)	Enzymatic hydrolysate: 378 µg/mL	-	-	[42]
			UF (<5 kDa molecular weight cut off (MWCO): 849 µg/mL			
			UF (1–5 kDa MWCO): 601 µg/mL			
			UF (<1 kDa MWCO): 150 µg/mL			
Date seed flour	Enzymatic hydrolysis with alcalase, flavourzyme, thermolysin and their mixture	-	530 µg/mL^2^ (alcalase and thermolysin enzyme mixture)	Quadrupole orthogonal time-of-flight (QqTOF)-MS/MS hybrid tandem mass spectrometer (QqTOF-MS/MS)	2.06–116.8 kDa	[14]
Peanut	Enzymatic hydrolysis with alcalase	UF (10kDa cut off), SEC	44.4 μg/mL^2^	Nano-LC-MS/MS	271 unique peptides 295–782 Da	[43]
Bitter melon seed	Enzymatic hydrolysis with thermolysin	UF (3 kDa cut off), HPLC	8.64 µM	LC-MS/MS	Val-Ser-Gly-Ala-Gly-Arg-Tyr 708 Da	[26]
Pea	*Lactobacillus plantarum* fermentation	SEC (Sephadex G-10), HPLC	64.04 µM	LC-MS/MS	Lys-Glu-Asp-Asp-Glu-Glu-Glu-Glu-Gln-Glu-Glu-Glu 1593.58 Da	[21]
Spinach	Enzymatic hydrolysis with pepsin-pancreatin	RP-HPLC	Fr 1: 4.2 µM	Protein sequencer	Fr 1: Ile-Ala-Tyr-Lys-Pro-Ala-Gly	[27]
			Fr 2: 2.1 µM		Fr 2: Met-Arg-Trp-Arg-Asp	
			Fr 3: 0.6 µM		Fr 3: Met-Arg-Trp	
			Fr 4: 0.38 µM		Fr 4: Leu-Arg-Ile-Pro-Val-Ala	
Cherry subproduct	Enzymatic hydrolysis with alcalase, flavourzyme and thermolysin	UF (3 and 5 kDa cut-off)	310 µg/mL^2^ (thermolysin hydrolyzate)	RP-HPLC-Q-TOF-MS	21 different peptides	[35]
Hemp seed	Enzymatic hydrolysis with alcalase, pepsin, papain and pepsin-pancreatin	SEC	16–228 µg/mL		300–9560 Da	[44]

^1^ Fr: Fraction; ^2^ IC_50_ value of the most potent ACE-inhibitory peptide; - not reported.
